# Cardiac Diagnostic Feature and Demographic Identification (CDF-DI): An IoT Enabled Healthcare Framework Using Machine Learning

**DOI:** 10.3390/s21196584

**Published:** 2021-10-01

**Authors:** Deepak Kumar, Chaman Verma, Sanjay Dahiya, Pradeep Kumar Singh, Maria Simona Raboaca, Zoltán Illés, Brijesh Bakariya

**Affiliations:** 1 Apex Institute of Technology, Chandigarh University, Mohali 140413, Punjab, India; Dr.d.k.mehta81@gmail.com; 2 Department of Media and Educational Informatics, Faculty of Informatics, Eötvös Loránd University, 1053 Budapest, Hungary; illes@inf.elte.hu; 3 Department of Computer Science and Engineering, Ch. Devi Lal State Institute of Engineering & Technology, Sirsa 125077, Haryana, India; sanjaydahiyakkr@gmail.com; 4 Department of Computer Science, KIET Group of Institutions, Ghaziabad 201206, Uttar Pradesh, India; 5 ICSI Energy, National Research and Development Institute for Cryogenic and Isotopic Technologies, 240050 Ramnicu Valcea, Romania; 6 Faculty of Electrical Engineering and Computer Science, “Stefan cel Mare” University of Suceava, 720229 Suceava, Romania; 7 Technical University of Cluj-Napoca, 400114 Cluj-Napoca, Romania; 8 Doctoral School Polytechnic University of Bucharest, 061071 Bucharest, Romania; 9 Department of Computer Application, I.K. Gujral Punjab Technical University, Jalandhar 144603, Punjab, India; dr.brijeshbakariya@ptu.ac.in

**Keywords:** cardiac disease, feature selection, multicollinearity, machine learning, IoT

## Abstract

The incidence of cardiovascular diseases and cardiovascular burden (the number of deaths) are continuously rising worldwide. Heart disease leads to heart failure (HF) in affected patients. Therefore any additional aid to current medical support systems is crucial for the clinician to forecast the survival status for these patients. The collaborative use of machine learning and IoT devices has become very important in today’s intelligent healthcare systems. This paper presents a Public Key Infrastructure (PKI) secured IoT enabled framework entitled Cardiac Diagnostic Feature and Demographic Identification (CDF-DI) systems with significant Models that recognize several Cardiac disease features related to HF. To achieve this goal, we used statistical and machine learning techniques to analyze the Cardiac secondary dataset. The Elevated Serum Creatinine (SC) levels and Serum Sodium (SS) could cause renal problems and are well established in HF patients. The Mann Whitney U test found that SC and SS levels affected the survival status of patients (*p* < 0.05). Anemia, diabetes, and BP features had no significant impact on the SS and SC level in the patient (*p* > 0.05). The Cox regression model also found a significant association of age group with the survival status using follow-up months. Furthermore, the present study also proposed important features of Cardiac disease that identified the patient’s survival status, age group, and gender. The most prominent algorithm was the Random Forest (RF) suggesting five key features to determine the survival status of the patient with an accuracy of 96%: Follow-up months, SC, Ejection Fraction (EF), Creatinine Phosphokinase (CPK), and platelets. Additionally, the RF selected five prominent features (smoking habits, CPK, platelets, follow-up month, and SC) in recognition of gender with an accuracy of 94%. Moreover, the five vital features such as CPK, SC, follow-up month, platelets, and EF were found to be significant predictors for the patient’s age group with an accuracy of 96%. The Kaplan Meier plot revealed that mortality was high in the extremely old age group ($\chi^{2}$ (1) = 8.565). The recommended features have possible effects on clinical practice and would be supportive aid to the existing medical support system to identify the possibility of the survival status of the heart patient. The doctor should primarily concentrate on the follow-up month, SC, EF, CPK, and platelet count for the patient’s survival in the situation.

## 1. Introduction and Related Work

An IoT and wearable monitoring systems are two emerging technologies expected to have a wide range of applications in healthcare. The integration of IoT aspects into medical devices improved the quality and effectiveness of the healthcare industry service. IoT incorporation into the health sector has led researchers worldwide to build intelligent applications such as mobile health care, healthcare suggestions, and competent healthcare systems. Smart wearable gadgets can be used for collecting patients’ health condition data, e.g., fructose levels, pulse rate, BP levels, and these data can be tracked constantly via wearable device sensors and transmitted to smartphones [1]. The sensor nodes of the Electrocardiogram can be connected to the IoT network, and the plug and play feature can be used to back up [2]. The collected data is stored on the cloud server via IoT technologies. It allows remote access to both real-time and historical data. IoT gadgets will offer a healthy life at a lesser price. To monitor patients’ heart rate, Abdel-Basset et al. [3] presented the IoT-based framework with computer-supporting diagnostics for obtaining real-time data. The collected body sensor data was taken from users’ mobile via Bluetooth technology and transmitted to the cloud. Kumar et al. [4] recommended a scalable three-tier architecture. Tier 1 was responsible for compiling the wearable sensor IoT device data. Apache HBase was used by Tier 2 to efficiently save the wearable IoT sensor data in a cloud environment. Tier 3 then used Apache Mahout to set out a cardiovascular logistics-based prediction system. The Cloud and IoT-based mobile healthcare application with fuzzy temporal neural classifier were developed to monitor and diagnose serious illnesses [5]. S. J. Park et al. [6] proposed an IoT-based consumer stroke prediction system to predict the disorder and healthy gait with more than 95% accuracy given by the C5.0 model. Further, the same author identified the stroke based on IoT sensors to support elderly drivers while driving [7].

Usually, the symptoms of cardiovascular disease (CVD) differ by gender in the patients. For example, a male patient is more likely to experience chest pain than a female patient. It was reported that female heart patients suffer from nausea, excessive exhaustion, and shortness of breath [8]. Researchers have been experimenting with a range of strategies to predict Heart Disease (HD). Angiography is the most precise and effective tool for predicting cardiac artery disease [9]. Still, it is costly, making it out of reach for low-income families, so scientists and researchers are looking for other ways to predict the outcome with detection of precise causes.

SS is routinely measured to determine electrolyte, acid-base, and water balance, as well as renal functionality in our body. The ideal range of the SS is 135–147 mmol/L. The leading cause of HF is higher fluid retention due to high sodium intake in the body. That’s why a sodium-free diet is recommended for the general public to avoid the HF disease, and also it was found to be linked with BP and hypertension [10,11,12]. Moreover, the sodium intake significantly impacts the renin, aldosterone, noradrenaline, adrenaline, cholesterol, and triglycerides [13]. Mohammed W Akhtar et al. [14] found that the renal insufficiency (abnormality in SC) in HF patients also increased five times more risk of death. Several studies had identified and supported the close connection between hypertension and dietary sodium intake. The blood sugar levels in people with diabetes could rise to dangerously high levels, causing health problems such as renal insufficiency [15]. Abede Tamrat et al. [16] found significant differences among hemoglobin, creatinine, and salt levels of anemic and non-anemic levels of patients.

Frequent Smoking could be a cause of atherosclerotic CVD [17]. Huxley RR et al. [18] noticed more HF among the young chain-smoker in the south Asian small group of study, and the authors also verified the sexual disparity among them.

Machine learning plays a critical role in every field of life such as medicine, engineering, industry, and education. Several popular machine learning algorithms are available today to solve the complexity and non-linear interaction between various variables. Researchers are encouraged to use machine learning algorithms in healthcare to analyze the data and make precise diagnostic decisions. Multiple studies have been done with different classification algorithms in health analytic to identify the CVD in patients [19]. Old age people were vulnerable to HD due to the degradation of cardiovascular functionality in their bodies [20,21]. According to the American health association, a total of 86% CVD patients were found suffering from the CVD [22]. The cox regression model had been used for the assessing the HF patient’s survival and reported a 32% mortality rate due to CVD. In addition, it was also found that EF, age, creatinine (creatinine > 1.5 renal dysfunction), sodium, anemia, and BP had a significant mortality rate. Smoking and diabetes were not found significant toward HF death. Sagar B. Dugani et al. [23] found that women were more vulnerable to cardiac disease, and risk profiles changed by age at the start of diabetes.

Further, the age group was predicted in the cancer patients using artificial neural network (ANN) with the accuracy of 59.09%. Alcohol abuse, Industrial hazard, estrogen exposure, and papillomavirus were significant key features with the help of the recursive feature elimination technique [24]. Adam S. Vaughan et al. [25] concluded that the mortality of HD decreased in all age groups (35–44, 45–54, and 65–74) except between 55–64 years of age. Sagar B. Dugani et al. [23] found diabetes and insulin resistance, together with hypertension, obesity, and smoking, as the most potent risk factors for the HD. Despite higher morbidity, it had been reported that women consistently had a lower rate of mortality than men in the assessment of death rate related to CVD [26]. Older females were confirmed to have a higher risk of CVD than age-matched men [27,28].

Villa et al. [29] examined the impact of gender on HD patients and found that women were generally safe from CVD before menopause, but their risk increased dramatically after menopause. Edward Korot et al. [30] used the auto ML model with the images of the retinal fungus and predicted the gender from the UK Biobank dataset with an accuracy of 88.8%. SVM showed promise as the best classifier with LOSSCV hyper-parameter tuning in identifying the HD with an accuracy of 92.37% [31]. Davide Chicco et al. [32] predicted a patient’s survival with two most significant key features (EF and SC) using Matthew Correlation Coefficient (MCC) of 0.61, and accuracy of 83.8%. ABID ISHAQ et al. [33] also designed and developed patient’s survival classification but with Synthetic Minority Oversampling (SMOTE) technique due to the existence of imbalance in the target variable and obtained accuracy of 92.62% with the ETC classifier.

The structure of the present paper is divided into thirteen sections. Section 2 frames the problem statement of the research. Section 3 outlined the major contribution and the significance of the present research. Section 4 elaborated the state-of-the-art research objectives, design and methods. Section 5 explained the machine learning Models with Hyperparameter tuning. Section 6 presented the encryption and remote access. Section 7 presented a proposed simulation setup for the present research. Section 8 focused on the basics of applied machine learning algorithms. Section 9 debated the on the various performance evaluation metrics. Section 10 reflected the results of seven experiments. Section 11 discussed the findings of the experiments. Section 12 presented the limitation of the study. Section 13 concluded the real crux of the extant research with the future proceeding.

## 2. Problem Statement

The IoT-based health monitoring system seems promising to reduce the death rate and economic percentages of HF expenditures. There is always a high demand for an efficient system that can carry out intelligent data analysis. Unfortunately, few existing IoT diabetic surveillance types of research focused on the improving in the system response performance, but these lack the predictive analysis [34,35].

Data consumers can avoid the high expenses of local storage and management by outsourcing their data to the cloud, but the users’ concerns about sensitive data privacy and security in the cloud are legitimate given the cloud servers’ lack of trust and the fact that outsourced data may contain sensitive information. Sensitive data can be encrypted before being transferred to the cloud to mitigate this issue. We can save the encrypted medical data in the cloud to prevent patient information from being leaked and later can be used in processing after decryption [36]. We used a standard public key encryption method in which the data is encrypted using the public key method but only owner can decrypt the data with his/her private key. A growing number of researchers have been working on public key encryption techniques to achieve the various security related functionalities.

Doukas C. et al. [37] proposed a IoT enabled Cloud-based system in which data were acquired by wearable devices such as biosignals, ambient temperature etc. that were in turn then passed to a gateway using established IoT communication mechanisms and subsequently to Cloud infrastructure. The gateway was responsible for capturing the signals and patient data and after that apply user access control as well as security transmission mechanisms by applying data encryption.

Over the last ten years, a theme has emerged for confirming the study of gender-based disparities in Coronary Heart Disease (CHD) as a foundation for clinical initiatives to enhance women’s outcomes [38,39]. It was found that the women were more vulnerable to CVD and SC related problems [40,41]. Therefore, it is imperative to study SC, SS, and gender-related features and to find the significant impact of smoking, BP, and diabetes. The Cox regression method for survival analysis may be employed to examine the effect of various existing demographic features on time-specific occurrence. New methods for accurate identification of CVD and other features are still needed to address these issues. Prediction accuracy without using SMOTE and further related performance enhancement is a significant challenge and gap of the research. Therefore, there is a need to improve and extend analytical work on other aspects of HD with IoT technology. Table 1 shows the comparative analysis of the current versus extant researches towards HD.

Ahmad T. et al. [42] used Cox regression model to predict the mortality. Breiman predicted survival– status with RF using SMOTE [43]. Chico D. [32] predicted Survival-Status on the same dataset. The accuracy was 74%, and it needs another hyperparameter tuning to improve the results. The Cox regression model can identify the specific age group of HF patients that is more vulnerable to mortality. In addition, statistical methods are appropriate to determine the impact of patient’s SC and SS on health-related complications such as diabetes, anemia, High BP, and the effect can be measured on the survival status.

These significant cardiac models need to be transformed into the IoT enabled framework to automate the impact and predictive identification system to develop an intelligent health care system for cardiac patients.

## 3. Contribution and Significance

In our research approach, we used statistical verification techniques to test significant features of CVD patients like SC, SS, and other relevant elements. Additionally, the present research proposed a secured IoT enabled framework based on statistical methods, machine learning techniques, Raspberry-Pi4, cloud-based Azure server, database, wearable sensor and public key encryption techniques. The relevant cardiac features (SS, SC, EF, platelets, and CPK) are also proposed in the framework. The summarized contribution of the present work is as follows:

- The multi-collinearity removal feature is applied to enhance the accuracy of all predictive algorithms for patient’s demography with selected features. Additional, a 10-fold CV method found the best generalization to a robust predictive model among eight machine learning classifiers based on MCC, F1-Score, and accuracy ranking performance metrics.
- The model also facilitates verifying the impact of patients’ complication levels like anemia, BP levels, diabetes levels towards SC and SS using Mann-Whitney non-parametric test. Further, gender and smoking level association are also verified with $\chi^{2}$ statistics.
- The proposed model also used the Survival analysis tool for ascertaining the impact of age group levels on the Survival-Status levels variable.
- This model resolved security issues through the digital certificates and PKI data encryption to ensure the security of patients’ data.
- During unprecedented time of Covid-19 pandemic, the presented IoT framework has an important utility for the patients in their self-isolation or self-quarantine. They can also send their daily health symptoms to their doctors via their IoT wearable devices. Therefore, the existing health system can also be improved and rapid with the present research.

The major significance of the research is summarised below:

- The present IoT based framework can be helpful in decision-making method that accurately predicts patient’s demography like age group (Adult and Very Old), Gender (Woman/Man) and Survival-Status (Alive/Dead) of cardiac patients.
- The selected significant features such as SS, SC, EF, platelets, and CPK might be helpful to cardiac doctors to diagnose their patients.

## 4. Materials and Methods

### 4.1. Objectives

After comprehensive review on existing studies with a focus on current deficiencies, we framed seven objectives to address a significant research gap. Out of seven, the first four required statistical analysis, and the rest of three need predictive analysis with machine learning.

- **Objective 1**: To explore the impact of SC and SS on the Survival-Status level of the patient.
  1. H_01: *No significant difference between Alive and Dead towards SC and SS*.
- **Objective 2**: To explore an impact of SC and SS on anemia, diabetes, and High BP levels of the patients.
  1. H_02a: *No significant difference between non-anemic and anemic levels towards SC and SS.*
  2. H_02b: *No significant difference between non-diabetic and diabetic levels towards SC and SS.*
  3. H_02c: *No significant difference between Normal BP and High BP towards SC and SS.*
- **Objective 3**: To explore the association of gender and smoking habit of the patient.
  1. H_03: *No significant association between gender with smoking habits*.
- **Objective 4**: To explore the impact of age group on the survival-Status levels (censored/Dead) of the patient.
  1. H_04: *No significant association between Age-Group levels and Survival-Status levels*.
- **Objective 5**: To identify the Age-group of HF patient based on significant features.
- **Objective 6**: To recognize the gender of HF patient based on significant features.
- **Objective 7**: To predict the HF patient’s Survival-Status levels based on the significant features.

### 4.2. IoT Enabled CDF-DI Framework

**Figure 1:**
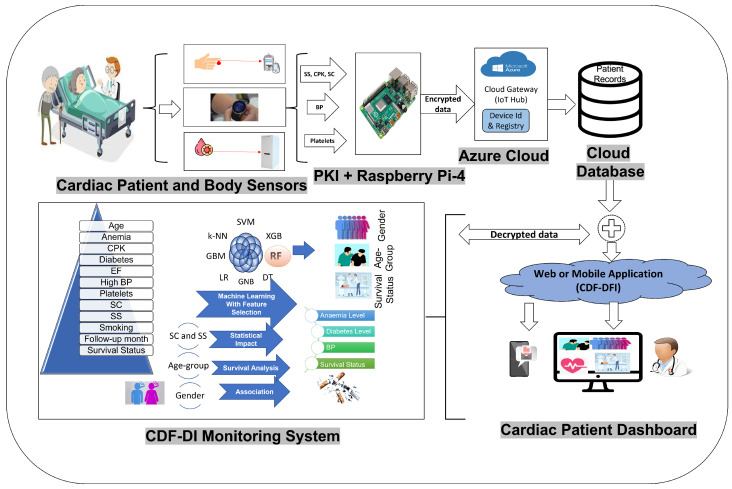
Conceptual Design of CDF-DI integration with IoT Framework.

Figure 1 showed the proposed framework with six primary components: Health wearable sensors devices, Raspberry Pi-4 with PKI module, Azure IoT Hub, Azure Cloud, UCI data repository data sets, CDF-DI Monitoring System, Cardiac patient dashboard, and mobile for getting any update.

Here, wearable health sensors devices are IoT devices. These gadgets can help in collecting the patients’ medical information from remote regions. Medical data are obtained with IoT instruments attached to the human body. Azure IoT hub is collecting the sensor data with its registered Raspberry Pi microcomputer system with cryptography module for privacy and security of data. Azure IoT hub, in its turn, send data to its cloud for storage. The UCI HF dataset is also used here for training and validating the CDF-DI monitoring system. All of these datasets (Sensor data and UCI dataset) have been uploaded to a cloud database. Finally, the CDF-DI monitoring system performs statistical and predictive analysis. The final prediction analysis is displayed to the doctor for information on the cardiac patient dashboard.

There are three phases in the proposed Cloud and IoT-based health care system. The essential data is collected from IoT devices, the UCI repository, and medical history data in the first stage. Phase two is used to store collected records on cloud databases safely. Phase three is responsible for the prediction and diagnosis of the condition. Phase-3 has sub modules which predict the Gender, Age-Group, and Survival-Status of patients utilizing various machine learning classification algorithms (RF, XGB, SVM, DT, GNB, GBM, LR, and k-NN). The second submodule is responsible for verifying the impact of SC and SS on the patients’ complications like anemia, diabetes, BP, and Survival-Status. The survival-Analysis approach is also helping to asess the mortality outcome for a specific patients’ age group in the CDF-DI monitoring system. Another sub-module verifies the association between Gender and smoking level of HF patients.

In addition to these functionalities, the system will also send all notification updates to the doctors and patients family about the status of their patients to the registered mobile numbers and also sound alarms when the patient’s body metrics deviate from the normal benchmark [44].

### 4.3. Work Flow Diagram

**Figure 2:**
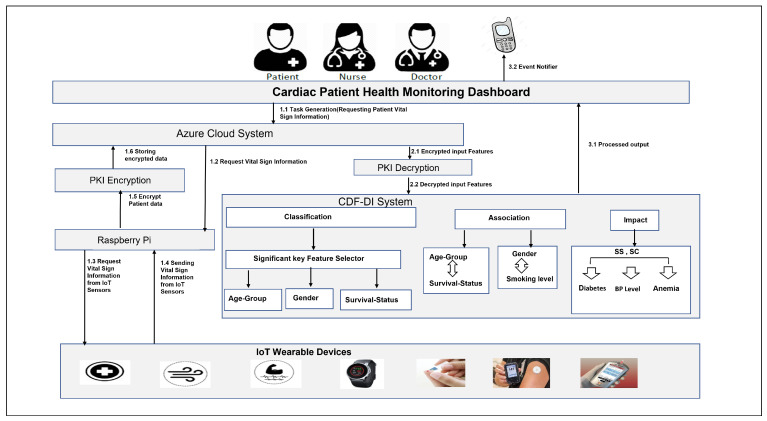
Workflow of IoT Based CDF-DI Framework.

Figure 2 visualizes the workflow of the proposed IoT enabled CDF-DI healthcare framework. The presented CDF-DI monitoring system provides three services: Classification, association, and impact identification with cloud-based edge technology. It provides several significant features to classify the patients’ survival status, age group, and gender. It supports an association of gender with smoking level and age group with survival status. Additionally, it explored the impact of SC and SC on the patients’ health complications levels. The participants like patients, nurses, and doctors can query to avail these services provided from their mobile and computers remotely. The generated queries shall be forwarded to the Azure cloud server, and handled with the Raspberry-Pi to collect patients’ data through its wearable IoT devices. Now, sensitive information of patients can be stored on a cloud server using the PKI encryption technique. These encrypted data will be decrypted with the private key and entered into the CDF-DI system to avail the desired service.

### 4.4. Dataset Description

This research used HF clinical dataset obtained from the UCI Machine Learning repository [45,46]. The dataset has a total of 13 features with 299 records of heart patients. The data are collected during the patients’ follow-up months. There are 194 men and 105 women among the 299 patients. The average follow-up period was 130 days, ranging from 4 to 285 days. Renal insufficiency is indicated by an SC level which is higher than the average level (1.5). There are no missing values. Table 2 shows the description (narrative, ranges, measurement units). Dataset has six categorical (binary) variables. The age of the patients are lies between 40 to 95, with a mean value of 60.83.

**Figure 3:**
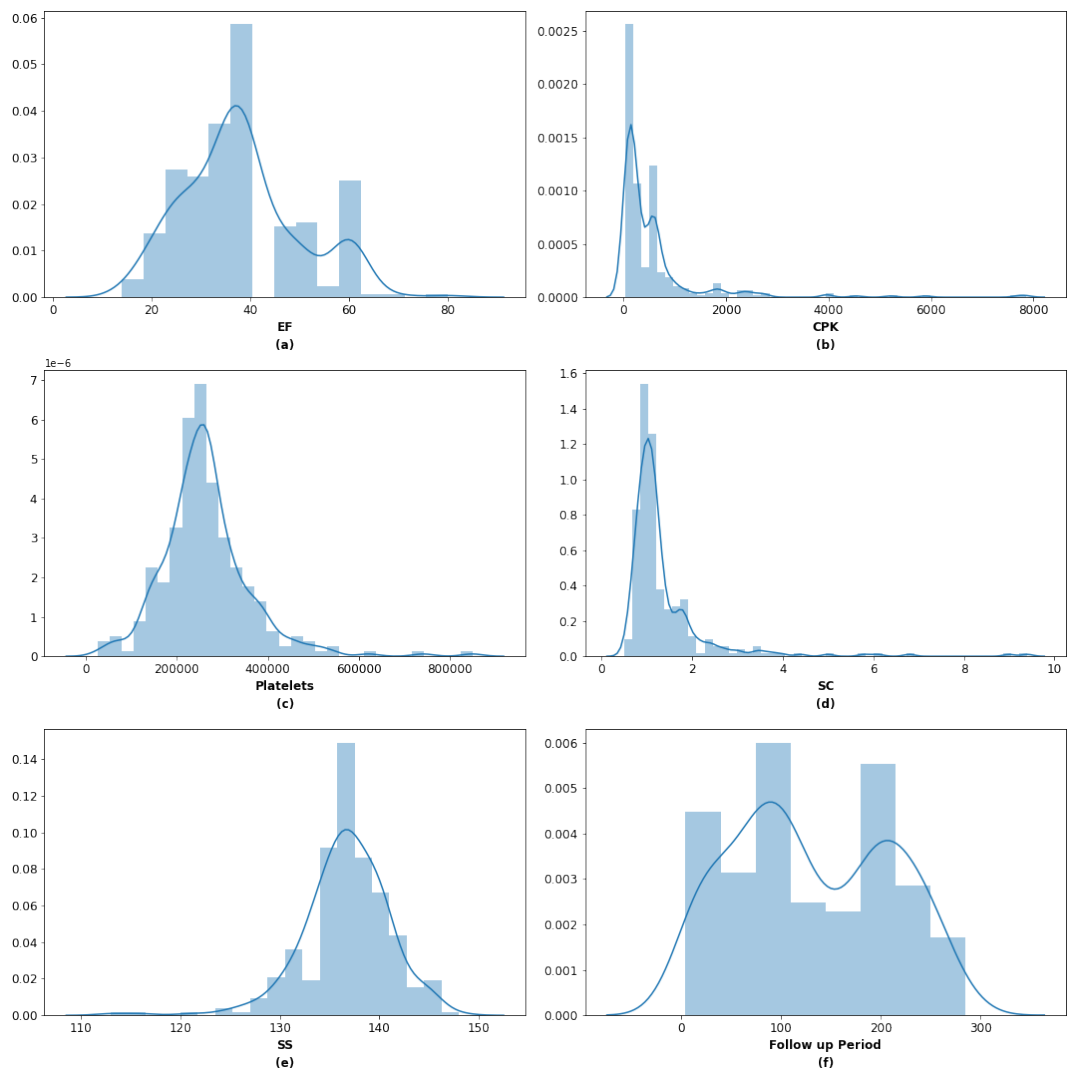
Dataset Distribution (**a**) EF, (**b**) CPK, (**c**) C-platelets, (**d**) SC, (**e**) SS, (**f**) Follow-up.

Figure 3 shows the distribution plot of metric variables available in the dataset. Figure 3a illustrates that non-normal EF distribution (*p* < 0.05) with mean of 38.08. Figure 3b displays the non-Normal distribution of the CPK with a mean of 581.84 with 251.49 kurtosis and 4.48 skewness. Figure 3c visualizes the non-Normal distribution, and it has a mean of 263,358.03 with 6.21 kurtosis and 1.46 skewness. Figure 3d depicts a non-normal distribution of SC, and its mean was 1.40. The kurtosis and skewness are 25.83 and 4.46, respectively. Figure 3e graphs the non-normal distribution of SS having a mean of 136.36 with the Kurtosis of 25.83 and skewness of 4.46. Figure 3f displays, and it is evident that the SS has non-normal distribution with 136.63 means. The lower bound and upper bound are 136.12 and 137.13, respectively, with 4.41 SD. Patients’ Follow-up months distribution plot is displayed in Figure 3f. From day 4 to day 285, the follow-up month mean is 130.26 found. The absence of normality is also there with 77.61 SD.

### 4.5. Preprocessing

Variance inflation factor (VIF) is a measure of the amount of multicollinearity in explanatory variables. The highest VIF leads to the issue of multicollinearity. Usually, its score should be less than 10 [47]. Therefore, multicollinearity is identified in SS, Age, EF features of the dataset. To remove it, a backward elimination method is used for feature selection. Tests for correlation and multicollinearity among features were employed using the VIF. After the removal of only two features: SS and Age, the VIF scores are found significant. Equation (1) shows the estimation of the VIF value of an attribute a∈A is determined from a dataset D = (A, X) using a standard linear regression. Where *R* is a regression coefficient of determination.

(1) $$VIF\left(a\right)=\frac{1}{1-R^{2}}$$

Further, to classify the age group of the patients towards survival in HF, the “Age group” has been derived as a novel feature from the existing age feature. It has been transformed using binning (bucketing) into two categories: Adult and very old. The encoding of Adult and very old has been set to 0 and 1, respectively. After bucketing, 129 patients belong to an adult, and 170 relate in Very Old categories. Table 3 shows the significant features after applied VIF with backward elimination. The model has considerable multicollinearity based on gender and age group as target features (VIF > 10).

Z-score is one of the most significant standardization procedures that may be carried out by subtracting the mean and dividing the standard deviation for each value of each feature [48]. Equation (2) shows the *z*-score calculation where the μ is the mean and σ is the SD of given continuous feature.

(2) $$z=\frac{x-\mu }{\sigma }$$

Standardization is applied on the dataset during preprocessing part of the SVC classifier in the 10-fold CV method of gender, age group, and survival-status prediction.

## 5. Models Hyperparameter Tuning

ML model’s efficiency can be increased on the given dataset by tuning hyperparameters. The method of hyperparameter selection is one of the most critical characteristics of ML models. Optimization of hyperparameters may be described as follows in Equation (3):

(3) $$x^{*}=arg.m_{x}\varepsilon_{X}f\left(x\right)$$

Here *f*(*x*) denotes the objective score to minimize the validation set errors, x* is the minimum score hyperparameters collection, and *x* may assume any domain value of *X*. The hyperparameters are tuned at 10-fold Grid Search CV to maximize the MCC score. The following listed parameters were utilized for ML models.

Hyperparameter search space is reflected in Table 4 for this research. In the RF classifier, the max sample parameter is managed if bootstrap = True (default), otherwise the entire dataset will be used to make a tree each [49]. For all proposed classifiers (Age-Group, Gender, Survival Prediction), Gini is used as a criterion and to measure the quality of a split, max-feature set to 7 for the best split, min-sample-leaf set to 2 as the least sample number necessary for the leaf node. The number of the decision tree is set by 50 using an n-estimators parameter. DT classifier, Gini with the max-features set as a log. The max depth and minimum sample split are set to 50. The SVM classifier regularization parameter is set as C (10) with a radial basis kernel. Gradient boosting classifier (GBM), a Learning rate of 0.001 reduces each tree’s contribution. The interaction of the input variables determines the best value is set as 3 with a number of an estimator (n_estimator) is 1000.

XGB, a scalable end-to-end tree boosting method commonly used by data scientists to achieve state-of-the-art results on a variety of machine learning challenges [50]. To avoid overfitting, step size shrinkage was used in the update (eta aka learning rate) set as 0.1 and gamma set as 0. Maximum depth of tree set as 6 and increase this result model become more complex with minimum child weight set as 4 with 200 trees in the forest (n_estimator). In k-NN, n_neighbour set as 3 with mahanntan metric and weight set as uniform. Newton-cg is set as a solver to handle the multi-class problem, and newton-cg handles only l2 penalty with regularization parameter C set as 1.0 in LR classifier.

## 6. Encryption and Remote Access

The IoT has the ability to connect a wide range of medical devices, sensors which helps the healthcare specialists to provide excellent medical services at a remote location. Therefore the safety for patients, healthcare costs, accessibility of healthcare services, and operational efficiency in the healthcare industry have all improved with the adoption of IoT techniques [51]. PKI can boost the trust significantly while exchanging the data in an unsafe environment, such as an IoT and over the cloud. Even If the hacker steals the public key, retrieving the private key would be computationally impossible because of the complexity involved in its calculation. In addition to secure data transmission, effective device authentication can be accomplished via PKI digital certificates. This idea can be implemented on Raspberry-pi device with public key cryptography technique on the IoT sensor collected data and further the encrypted data can be sent to the cloud storage. Further this CDF-DI analysis system can analyze this data after making the decryption of stored data with their private key.

The proposed remote smart healthcare support system would allow patients to be monitored for their health condition and to receive prescriptions from their doctors while they’re at remote location (home). Moreover, doctors can also carry out the diagnosis of ailments using the data collected remotely from the patient. An Android-based mobile application that connects with a web-based application allows for efficient patient-doctors dual real-time communication.

A Raspberry-pi microcontroller will perform the overall task of the system. IoT based wearable devices: BP monitoring, platelet counter, glucose monitoring, blood cretanine analyzers are connected to the Raspberry-pi for gathering and data transmission to Azure cloud and also to the mobile application for home control. Raspberry-pi has some digital pins can be used to relay the settings of the wearable devices [52].

## 7. Simulation Setup

An IoT-based proposed framework integrates various elements: Wearable sensors devices, Raspberry Pi device, MS-AZURE IoT hub (acting as a gateway) for transferring data to the cloud system. Along with patient identity, their follow-up months, age, and sex are maintained inside the system. In the absence of an instrument to assess EF, a pseudo-random number method generates 14 to 80. The BP parameter is measured using Omron HeartGuid-bp8000m, which provides cloud data and mobile telephone notifications. It is used for setting the High BP value when the input value becomes greater than the Normal level. Abbott’s FreeStyle Libre System monitors glucose levels. Anemia and smoking are also retrieved from the patient’s medical history records. The Raspberry Pi single-board computer [53] is used to capture and process the data. Table 5 lists the hardware required for this experiment.

## 8. Machine Learning Experiment Design

The present paper experimented on machine learning binary classifiers for prediction on given dataset using: DT [54], RF [43], k–NN [46], LR [55], XGB [56], GBM [57], GNB [58], Linear, Radial SVM [59]. Table 6 shows the employed machine learning classifiers. The experiment trained and tested with it 10-fold cross-validation using the grid search CV method. Each model was trained with a different hyper-parameter on the training set applied it to the validation set and then chose the model with the highest MCC to apply to the test set. In this experiment, we repeated experiments ten times for all classifiers and documented the highest result for MCC. We arranges the result table according to ranking based on MCC first, F1-Score (second), and then finally, results were arranged based on accuracy. Results were displayed on the theme of different metrics, different ranks. The three rankings we applied to the report yielded the same results, revealing intriguing features, when ranking based on MCC, F1-score, or the accuracy, the top classifier changes.

## 9. Performance Evaluation Metrics

Scientific research uses a variety of performance matrices to assess prediction accuracy [60,61,62]. In binary classification problems, accuracy and F1-Score derived from Confusion matrics (CMs) have employed metrics for performance evaluation. The proportion of actual negatives that were predicted as negatives is known as specificity (or True Negative Rate (TNR)) in Equation (4).

(4) $$TNR=\frac{\left(TN\right)}{\left(TN+FP\right)}$$

The recall or sensitivity in Equation (5) is a metric to detect the true positives instances in a model. It identified actual rates of patients with HF.

(5) $$Recall=\frac{\left(TP\right)}{\left(TP+FN\right)}$$

Precision is the ratio of True Positives to all Positives in its most primitive sense as described in Equation (6).

(6) $$Precision=\frac{\left(TP\right)}{\left(TP+FP\right)}$$

The ratio of the overall number of correct predictions to the total number of predictions is known as accuracy as described in Equation (7).

(7) $$Accuracy=\frac{\left(TP+TN\right)}{\left(TP+TN)+(FP+FN\right)}$$

The Harmonic Mean of Precision and Recall is the F1-Score as described in Equation (8). It helps to sustain stability between precision and recall.

(8) $$F1-Score=\frac{\left(2\times TP\right)}{2\times TP+FP+FN}$$

Equation (9) shows the Mathews Correlation Coefficient (*MCC*). It would be a more accurate statistical measure that only yields a high score if the prediction performed well in all four CMs groups (true positives, false negatives, true negatives, and false positives) despite imbalance dataset [63]. The *MCC* would produce a high score only if the binary predictor are able to correctly predict the majority of positive data instances and the majority of negative data instances while working with binary classification [64]. It has the worst value of −1 and the best value of +1.

(9) $$MCC=\frac{\left(TP\times TN)-(FP\times FN\right)}{\sqrt{\left(TP+FP)\times (TP+FN)\times (TN+FP)\times (TN+FN\right)}}$$

## 10. Experiments and Results

### 10.1. Experiment-1

This experiment was conducted to verify the significant differences between Alive (0) and Dead (1) patients towards their SC and SS level using non-parametric Mann–Whitney *U*-test. Table 7 shows the *U* test statistics with *p*-values for both SC and SS.

It was observed that Survived and Non-Survived patients’ SC were significantly different (*U* = 5298, *p* < 0.05). It was evident from the nonidentical mean rank of Survived and Non-Survived patients’ SC (μ rank: 128.10 & 196.31). Test results indicated a higher non-survived patient’s level than in survived patients.

In the case of SS, the difference in mean ranks (μ rank: 162.40 & 123.78) could also be observed. Thus, the SS of survived patients (μ rank: 162.40) was higher than non-survived patients (μ rank: 123.78). A Mann–Whitney *U* test indicated that this difference was statistically significant (*U* = 7226.50, *p* < 0.05).

**Figure 4:**
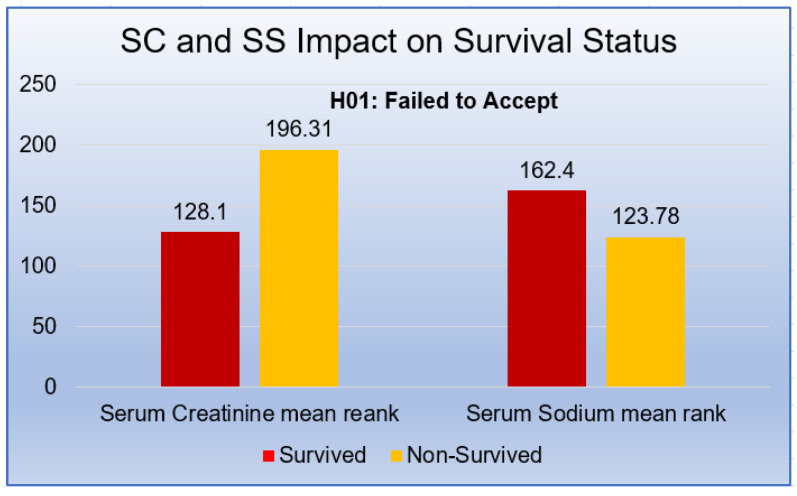
SC and SS Impact on Survival Status.

Figure 4 displayed the substantial differences in SC with mean rank (μ rank: 128.10 & 196.31) of survived and non-survived patients. Hence, it could be easily concluded that non-survived patients’ SC level was greater due to HF. However, in the case of SS, the reverse scenario could be observed. Non-survived patient’s SS level became low (μ rank: 123.78 & 162.40) when a patient suffered from HF. Therefore, this experiment results indicated a statistically significant impact of SC and SS on the Survival-Status of the patients, i.e., we rejected the null hypothesis H_01.

### 10.2. Experiment-2

The experiment was conducted to test the three null hypotheses (H_02-H_04) to verify the differences in SC and SS towards anemic, diabetic, and high BP levels. All the hypotheses were tested with a Mann Whitney *U* test.

Table 8 shows that the Mann–Whitney *U*-test statistics of related hypotheses: H_02_a, H_02_b and H_02_c experiments results. We found no significant *p*-values in anemic levels (Non-Anemic (0) and Anemic (1)), diabetic levels (Non-Diabetic (0), Diabetic (1)), and BP level (Non-BP (0), BP (1)) towards SC and SS.

The SC towards non-anemic (μ rank = 151.22) and anemic (μ rank = 148.40) patients were not significantly different, according to test results (*U* = 10,758, *p* > 0.05). Same scenarios could be noted in the SS w.r.t non-anemic (μ rank = 145.40), and anemic (μ rank = 156.06) levels were not also significantly different with (*U* = 10,183.50, *p* > 0.05). Therefore, findings recommended an insignificant *p*-values in the case of SC and SS. In SS, we failed to reject “H_02_a: No Significant difference between non-anemic and anemic levels towards SC and SS”.

Further, the SC towards non-diabetic (μ rank = 149.86) and diabetic (μ rank = 150.20) levels patients were not statistically significantly different (*U* = 10,850, *p* > 0.05). Same result patterns could be observed in the SS towards non-diabetic (μ rank = 154.03) and diabetic (μ rank = 144.38) level patients were not also different with (*U* = 10,173.50, *p* > 0.05) towards SS. Therefore, we also failed to reject the null hypothesis (H_02_b), i.e., there were no significant differences in diabetic and non-diabetic patients towards SC and SS.

Moreover, the SC in Non-BP (μ rank = 155.67) and BP (μ rank = 139.52) levels patients were not found fundamentally unique, as indicated by a *U* test statistical results (*U* = 9085, *p* > 0.05). Similar conduct could be noted in the SS towards BP level in non-BP (μ rank = 148.78), and BP (μ rank = 152.25) patients were not likewise unique with (*U* = 9948.50, *p* > 0.05) towards SS.

Therefore, we also failed to reject the null hypothesis (H_02_c): *“No significant difference between Normal BP and High BP towards SC and SS"*. There were no significant differences between Non-BP and BP patients towards SC and SS.

**Figure 5:**
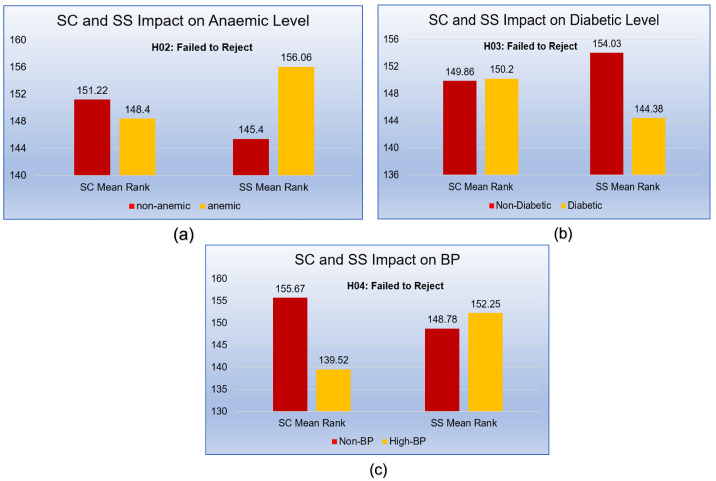
SC and SS Impact on (**a**) Anemic level, (**b**) Diabetic Level, (**c**) BP.

Figure 5a displays, the closest mean SC ranks towards anemic levels (μ = 151.22 > μ = 148.40). In the case of SS, non-anemic patients had a greater mean rankf SS as compared to the anemic patients (μ = 145.4 < μ = 156.06). Findings showed no significant result in the mean ranks of SS. (*p* > 0.05).

From Figure 5b, Diabetic patient’s mean rank of SC level was found greater than Non-diabetic patient’s mean rank (μ = 149.89 < μ = 150.20). conversely, non-diabetic patient’s mean rank of SS was found greater as compared to diabetic patient’s mean rank (μ = 154.03 > μ = 144.38). Unfortunately, the statistical test suggested an insignificant difference (*p* > 0.05).

Figure 5c reflects the greater difference in the SC’s mean ranks (μ = 155.67 > μ = 139.52), but in the case of SS, mean ranks difference seems close to BP patients (μ = 148.78 < μ = 152.25), but no statistically significant differences found towards SC and SS towards BP levels (*p* > 0.05).

### 10.3. Experiment-3

This experiment was performed to verify the association between gender and smoking levels of HD patients. The experiment used a non-parametric $\chi^{2}$ test to explore the association of gender level with smoking levels. A Cross-tab table was generated to investigate the association between two nominal variables.

Table 9 shows the cross-tab of observed values of gender and smoking levels, and Table 10 shows the expected values with $\chi^{2}$ of each cell. Residuals (Observed-Expected) are also marked in this table. A positive residual cell $\chi^{2}$ value means that the observed value is higher than expected. A negative cell residual $\chi^{2}$ value (e.g., −29.7) represents the observed cases are less than the expected number of cases.

Table 10 shows the most significant $\chi^{2}$ value 26.17 is found in the second cell. It is because the observed female smoker patients were 04, whereas 33.7 was expected. Therefore, the 2nd cell consists of a much larger number of expected cases than observed. This means that the number of observed female smoker patients was significantly less than expected. The second-largest $\chi^{2}$ value of 14.16 is located in the Male Smoking patient’s cell. However, we have noticed that this cell’s number of observed cases was significantly greater than expected (Observed = 92, Expected = 62.3). This indicates that a substantially higher number of male smokers was observed than what was actually expected. The third-largest cell $\chi^{2}$ value of 12.37 is located in a non-smoker females’ cell. The observed value of 101 and the predicted value of 71.30 for a non-smoker female were found. This means observed female non-smoker was significantly greater than expected. The last $\chi^{2}$ value was 6.70, in which expected non-male smokers (131.70) were considerably smaller than observed (102).

Further, It was obvious that the two groups were significantly associated (*p* < 0.05) with $\chi^{2}$ (1) = 59.45. Therefore, these findings suggested to reject the null hypothesis H_03 that no significant association of gender with smoking level.

### 10.4. Experiment-4

This experiment was conducted to explore the significant association of age group towards the survival-status levels using the Survival Analysis-Cox regression model tool. From the dataset, out of 299 patients, 129 were adults; 170 were from very old age group groups.

Table 11 shows a descriptive summary of age group towards survival-status levels in which 31 (24%) were from the adult age group and 65 (38.2%) from very old age group were found non-survived due to HF 98 (76%) adults and 105 (61.8%) very old age group patients were categorized as censored (study period was over and the patient has not experienced the event/Patient’s follow-up was lost).

Diabetes, platelets, gender, and smoking were found non-significant factors in the Cox regression model output in below Table 12. The negative age group coefficient values showed that CVD risk from CVD in “Adults” has lower than “Very Old”. The Age Group’s hazard ratio (HR) exp value (exp (−0.60) = 0.55) showed that 55 deaths happened out of every 100 deaths. These deaths were due to HD in which particular age group attribute level was played a significant role. Further results were illustrating the considerable part of CPK, anemia, EF, and high BP towards Survival-Status levels (*p* < 0.05).

**Figure 6:**
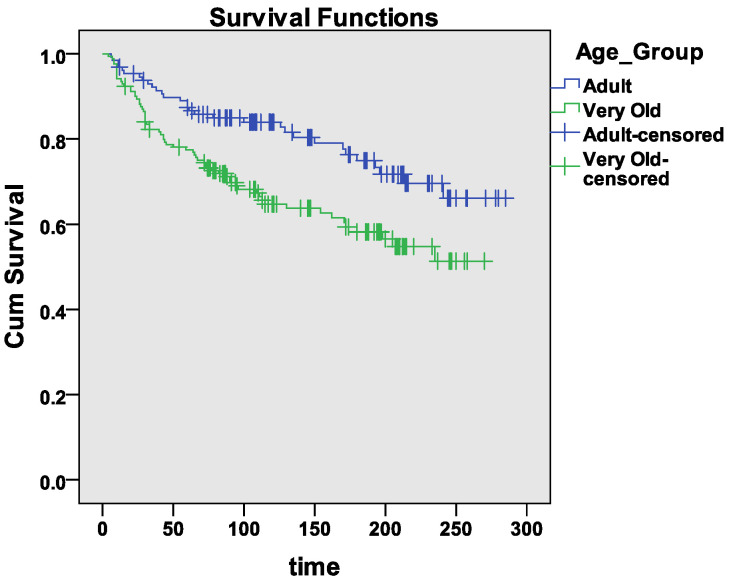
Kaplan Meier Curve of Follow-up months vs. Age Group Level.

Figure 6 demonstrates the survival function using Kaplan Meier Survival Curve. The x-axis represents the patients’ follow-up months, and the y-axis represents survival rate w.r.t to age group levels. The survival rate for the “Very Old” age group was lower than that of the “Adult” age group. It also indicated a statistically significant difference between the two levels with a log-rank *p*-value of 0.003 (*p* < 0.05) with $\chi^{2}$ (8.565), *df* = 1. The censored patients were highlighted with a cross on the curve. Therefore the experiment’s findings recommended rejecting the null hypothesis H_04 that there was no significant association between age group with Survival-Status levels.

### 10.5. Experiment-5

This experiment identified heart patient’s gender (Woman, Man) using various state-of-art classification algorithms as demonstrated in Table 13. Eight prediction algorithms (RF, GBM, DT, XGB, LR, SVM, k-NN, and GNB) were evaluated with the 10-fold stratified cross-validation technique and were compared all on six performance metrics: MCC, F1-ratio, Accuracy, and Precision and recall (TPR), and TNR (specificity).

Table 13 confirms that the RF has outperformed others in terms of highest MCC (+0.87) with SD 0.25, accuracy (0.94) with SD 0.11, and F1-Score (0.95) with 0.09 SD. Furthermore, the results also supported that the RF model achieved the highest TNR and TPR with 0.95 and 0.95 respectively. However, GNB model in terms of MCC, F1-Ratio and accuracy was found the worst performer with +0.06, 0.71, and 0.59, respectively. Moreover, in terms of recall, it was found that k-NN algorithm was the next performer after RF with 0.80 and in terms of specificity, GBM was found next performing classifier after RF with 0.68.

Gender classification algorithms’ confusion metrics (CM) displayed in Table 14. It can be noticed that the majority of the algorithms achieved a classification accuracy of more than 59%. It is clear from the results of CM that RF made the actual right highest prediction of 185 men and 96 women, and only 9 men and 9 women were wrongly predicted. The major misclassification occurred in the GNB model. Basically, 74 women were misclassified as men, and also the major misclassification found in 69 men, were wrongly predicted as women.

**Figure 7:**
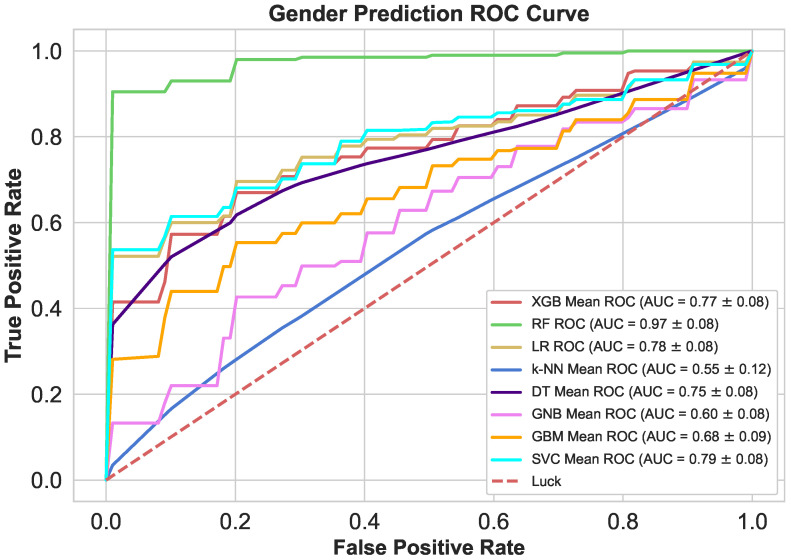
Gender classifiers’ ROC’s at dynamic thresholds.

Figure 7 displays the ROC curve drawn at 10-fold CV. It plots the True Positive Rate (TPR) against False Positive Rate (FPR) of all proposed algorithms at various threshold values. The AUC (area under the ROC curve) is another method for determining a classifier’s predictability. Its AUC value measures the classifier’s superiority, and the mean RF AUC (green color) 0.97 with SD of ±0.07 was found highest followed by the mean SVM AUC 0.79 with SD ± 0.05. The k-NN was the worst performer in the ROC curve with a mean k-NN AUC was 0.55 with an SD of 0.12.

**Figure 8:**
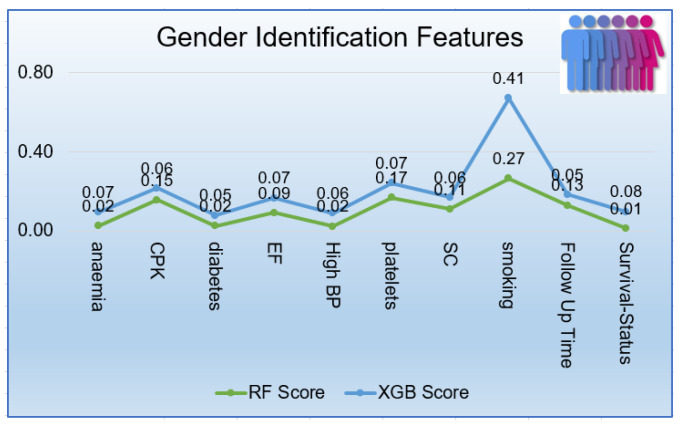
Gender identification Features.

The RF was the best performer in all terms of performance metrics. Therefore the RF must extract out important features by its inherent feature selection method. XGB was chosen as feature selection due to its gain method across all splits for feature extraction. As displayed in Figure 8, RF and XGB were used to perform the feature selection in the predicting of patient’s gender. RF Classifier considered smoking as first key predictor with value of 0.265,next platelets (0.167), CPK (0.155), SC (0.109), EF (0.091) during follow-up months (0.129). The XGB classifier also gave the same importance to Smoking with the value of 0.41.

### 10.6. Experiment-6

This experiment predicted the Age-Group (Adult, Very Old) of heart disease patients using various classification algorithms (Table 15). Here RF had the best performance in terms of MCC = +0.92 with SD 0.23, F1-score (0.96) with SD 0.11, and accuracy (0.96) with SD 0.11. The SVC classifier was the worst among all listed algorithms in terms of MCC = 0.02 with SD 0.17, but in terms of F1-Score, the k-NN classifier performed poorly (0.62 with SD 0.08 accuracy) followed in ranking by GNB classifier with 0.55 with 0.05 SD. Indeed, RF was outstanding in both the recalls (TP ratio = 0.97) with SD 0.07 and specificity (TN ratio = 0.95) with SD 0.16.

The Age-Group classifiers’ CMs are shown in Table 16 with eight classification algorithms. The majority of the predicting the age group algorithms have achieved classification accuracy greater than 55%. Among all specified algorithms, the RF classifiers’ CM TP VO (Very Old):165 and TN A (Adult):122 values were the highest among all. Further, only the XGB algorithm could find the next highest Adults (72) prediction, and only the GBM algorithm made the accurate prediction in very old (147) in Age Group next to RF model. However, a very substantial number of adult patients (107) were misclassified as Very Old in the GNB classifier. In contrast, many Very Old patients (62) were misclassified as adults in the k-NN classifier. Indeed RF classifier’s performance revealed the lowest misclassification rate with only seven adult patients misclassified as very old and only 5-very old patients wrongly predicted as adult patients.

**Figure 9:**
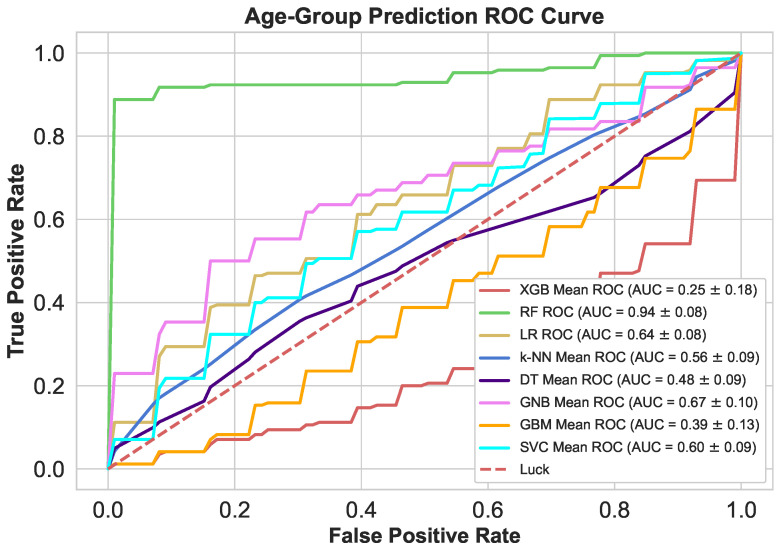
Age Group classifier’s ROC’s at dynamic thresholds.

As shown in Figure 9, the evaluated discriminator models performance spans from very low to good discriminatory levels (0.25 <= AUC <= 0.94). The top five discriminator (RF (0.94), GNB (0.67), LR (0.64), SVC (0.60) and k-NN (0.56)) based on AUC metrics. XGB and GBM models were found worst discriminator with 0.25 and 0.39 values in this experiment. The excellent discrimination made only by RF model.

**Figure 10:**
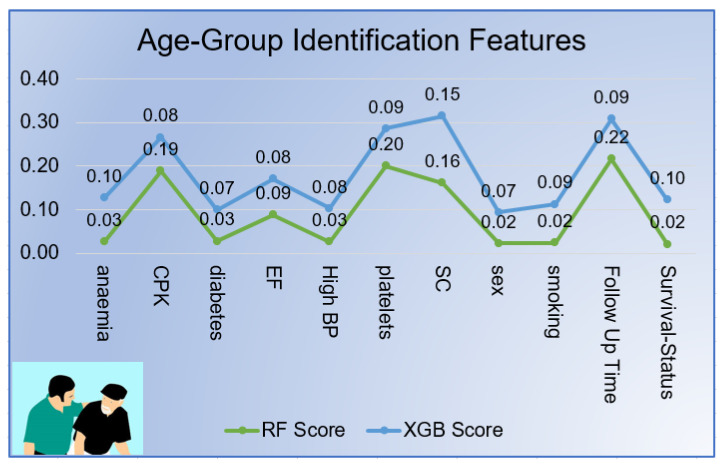
Patient Age group Features.

Figure 10 displays the most significant features that correctly identified the the age group of patients. The RF inherent feature selection technique found the platelets as highest key predictor with coefficient of 0.201 followed by CPK (0.189), SC (0.161), EF (0.088) and diabetes (0.027) during patients’ Follow-up months (0.216). It was also evident from the figure, the top significant features extracted by XGB classifier were SC (0.154), anemia (0.101), Survival-Status (0.101), smoking (0.088) and platelets (0.085) during follow-up months (0.093). Both classifier rank differently features to predict the Age group. CPK and platelets counts were considered as most significant features by both classifiers.

### 10.7. Experiment-7

This experiment identified the Survival-Status of patients using various classifiers as illustrated in Table 17. Findings recommended that we consider RF classifier as the best preforming model in terms of MCC (+0.91 with ±0.11 SD), in F1-score ranking (0.94 with ±0.07 SD) and accuracy ranking (0.96 with ± 0.06 SD). DT and XGB model followed in terms of MCC = +0.63. The k-NN classifier was the worst performer among all specified algorithms with MCC = +0.06, accuracy of 0.61, but in terms of F1-Score ranking, SVM-SVC were found to be as poor with 0.31. Indeed, the top MCC ranking classifier-RF model performed admirably in both recall terms (TP rate = 0.93) and specificity terms (TN rate = 0.98) respectively. The k-NN model was not able to predict a large percentage of patients correctly.

Table 18 represents all the CMs of survival-status classification algorithms. Here, TP (second row) represents the total number of right-predicted alive patients, and TN (first Row) represents the total number of right-predicted dead patients. Among all specified CMs, the RF classifiers’ CM displayed notable results with the highest Dead (89) and Alive (198). Only seven dead patients were wrongly predicted as alive and only 5 alive patients misclassified as dead by RF classifier compared to other classifiers. The k-NN algorithm has made the highest misclassification in the prediction of alive (49) and in dead (67).

**Figure 11:**
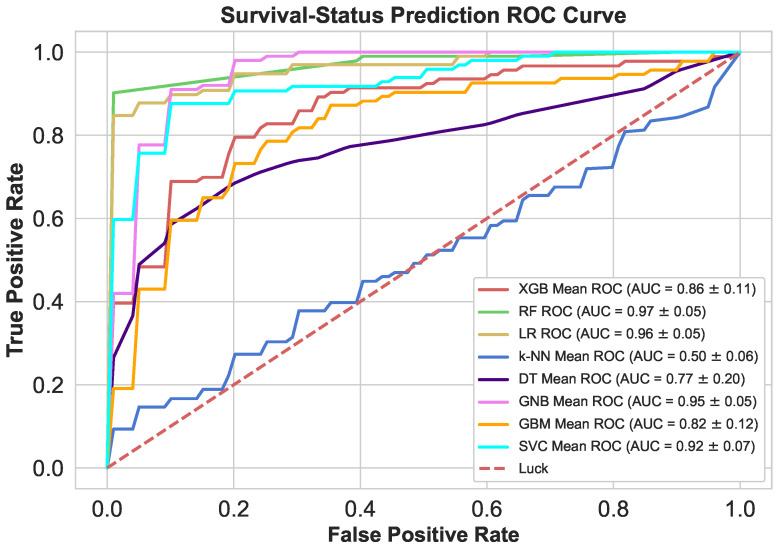
Survival Status classifier’s ROC’s at dynamic thresholds.

An ROC curve is created by plotting TPF (sensitivity) versus FPF (1-specificity) over a range of cut-offs. Figure 11 shows that the ROC curves with increasing discriminant capacity of diagnostic tests are gradually closer to the upper left hand corner. It can also be seen that the RF curve (AUC = 0.97) has a higher discriminating capacity than rest available curves.). The worst mean k-NN AUC (0.50) reflects very poor discriminative capability.

**Figure 12:**
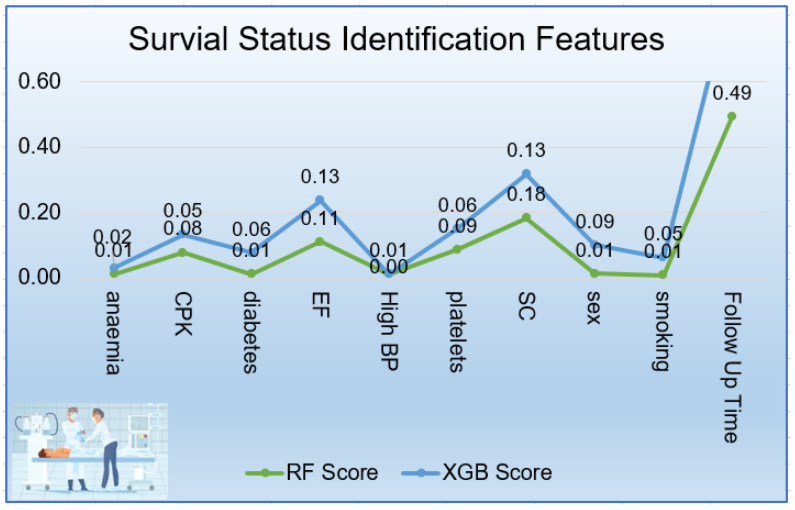
Patient Survival Features.

As seen in Figure 12, Follow-up months (0.492), SC (0.182), EF (0.110), platelets (0.086), CPK (0.077) and gender (0.012) were significant features to predict survival-status of patient. It was also obvious from visualization, the top significant features extracted by XGB classifier: SC (0.134), EF (0.127), gender (0.087), diabetes (0.065) and platelets (0.064) during follow-up months (0.396). Both classifiers considered SC and EF as the most significant features. but XGB treated the same gender at 3rd rank and RF treating the same at 5th rank.

## 11. Discussion

The significant *p*-value is critical for evaluating the hypothesis in statistical tests. To test the first two hypotheses, the Mann-Whitney *U* test was used in this study. It played a vital role due to the absence of normality in data. The authors had investigated the impact of SC and SS on the Survival-Status levels as well as on patients’ health complications levels such as anemia, diabetes, and high BP. The association between gender and smoking habit and the association between age group and survival-status were validated with $\chi^{2}$ and a cox-regression model.

The first null hypothesis, “H_01: No significant difference in Alive and Dead towards SC and SS”, was rejected (*p* < 0.05). There were statistically significant differences existed between the SC and SS w.r.t Survival-Status levels. The second null hypotheses “H_02a: No significant differences between non-anemic and anemic levels towards SC and SS”, “H_02b: No significant differences between non-diabetic and diabetic levels towards SC and SS”, and “H02c: No significant differences between non-BP and BP levels towards SC and SS” were all not statistically significant (*p* < 0.05). Furthermore, the patients’ gender and their smoking habits were significantly associated (*p* < 0.05). As a result, the third null hypothesis, “H_03: No significant relationship between gender and smoking level”, was rejected. During the study, it was found that actual Smoker female patients (04) were significantly less than expected (33.7) compared to non-smoker female patients. It was also observed that actual male smoker patients (92) were substantially more significant than expected (62.3) but not the same in male non-smoker patients. An actual male who was not accustomed to smoking habits (102) was found to be significantly less than expected (131.70) during follow-up months. The present study used also the cox regression model to explore the association between age group and survival-status levels and demonstrated a statistically significant association between these two attributes. Therefore “H_04: No significant association among age groups and Survival-Status level” was rejected (*p* < 0.05).

The study’s findings are self-evident: H01, the Survival-Status was linearly correlated with SC and SS (*p* < 0.05), supporting other author reports [12,13,14,42]. In the case of (H_02a, H_02b, and H_02c) refering to complications such as anemia, diabetes, and high blood pressure, SC and SS influences were not statistically significant (*p* > 0.05), contradicting autor reports [10,15] but supporting this author report [42]. The gender-smoking-habits association (H03) were significant (*p* < 0.05) supporting the article [17]. The age group Survival-Status association (H_04) finding was also statistically significant (*p* < 0.05) in contrast with the reports mentioned in reference [25] but supported by the reference [23].

The proposed CDF-DI was performed on a heart disease dataset and showed promising results compared to previous models in improving prediction accuracy. For comparison, we used eight state-of-the-art MLAs (GNB, LR, GBM, SVM, DT, XGB, k-NN, and RF) throughout the study that have an established track record for accuracy and efficiency in the research community. All models were subjected to 10-fold cross-validation, and six performance metrics were collected: Accuracy, precision, TPR, F1-measure, MCC, TNR. RF classifier was found superior in all mentioned performance ranking scores (MCC score ranking, F1-Score ranking, and accuracy score ranking) in all machine learning prediction goals.

The proposed model outperformed with RF machine learning model in predicting patients’ gender another model by obtaining accuracy up to 94%, and 95% in all metrics, i.e., precision, TPR, and F1-Score, respectively. In addition, the proposed CDF-DI model had the highest MCC values, up to 0.87, proving its superiority over other models. Furthermore, the proposed model had the lowest FPR and the highest TNR by up to 9% and 91%, respectively. The suggested model’s low FPR and high TNR values demonstrate the CDF-DI model’s capacity to reduce miss rates and improve classification accuracy for both negative and positive subjects. Table 13 displays the comprehensive performance findings for predicting patients ‘gender. GNB was the worst performer in MCC, F1, and Accuracy score ranking with 0.06, 71%, and 59% respectively.

Further, the predicting age group of patients had displayed encouraging results with the RF model. The proposed model was found as the best with RF in all key performance criteria, such as precision, TPR, F1-Score, and accuracy, 95%, 97%, 96%, and 96%, respectively. GNB model found the worst performing model in terms of MCC ranking and accuracy ranking with 0.02 and 55% scores, respectively. The k-NN model was worst in F1-Score ranking during the prediction of age group of patients with 62% scores. Table 19 summarizes all of the performance findings.

Furthermore, the RF model had also shown promising results in predicting the Survival-Status of patients. Performance metrics (precision, TPR, F1-Score, accuracy, MCC, and TNR) were found better with RF by up to 95%, 94%, 96%, 91%, and 97%, respectively. The k-NN model proved significant with MCC and accuracy score ranking, and in F1-Score ranking, the SVM model found a weak model. Table 17 summarizes all of the performance findings.

This is incredibly encouraging for hospital settings: Even if several laboratory test data and health conditions were absent from a patient’s electronic health record, doctors could still predict patient survival by evaluating the EF, SC, and CPK values alone. The present research also yielded several intriguing outcomes that varied from the findings of the same dataset study [58]. Davide Chicco et al. identified EF, SC, age, CPK, and gender chosen as the top five features for predicting Survival-Status while Tanvir Ahmad et al. [14] also identified age, SC, High BP, EF, and anemia as top essential features. This study found SC, EF, platelets, CPK, and gender as important features which play an essential role in predicting Survival-Status with RF Classifier as depicted in Figure 12. We found EF at 2nd position and also found platelets as an essential feature which the previous study had not found. The present paper also improves the accuracy, F1-Score, and MCC by 0.22, 0.39, and 0.53 respectively in RF classifier and other models as depicted in Table 19.

The experiment results displayed that the supervised machine learning models performed the best role in efficiently predicting the heart failure patients’ age group and gender. Tree-based algorithms performed well on the imbalanced dataset using the 10-fold cross-validation method. As it was displayed in Figure 10, RF identified CPK, SC, follow-up month, platelets, and EF as significant features while predicting the patients’ age group (adult, very old). Moreover, the RF classifier identified smoking, CPK, platelets, follow-up month, and SC. These methods became beneficial in-patient care because doctors can predict a patient’s age group based on only five significant characteristics. RF and XGB commonly extracted out SC and EF as crucial features in predicting the age group target variable. Moreover, smoking, CPK, and platelets were found vital features in predicting the patient gender common in RF and XGB.

The top five input features playing a vital role in predicting Survival-Status were presented in Figure 12. The top 5 features selected by RF and XGB inbuilt feature selection techniques were follow-up month, SC, EF, CPK, and anemia. The RF and XGB feature selection approach had lot in common. Follow-up month had the highest ranking (0.49), whereas anemia had a lower score (0.01) extracted by the RF feature selector. The XGB algorithm also treated the follow-up month feature as the most important (0.40) and gave the lowest rank to the the anemia (0.02). our findings suggest a special attention at the patients’ SC, EF, platelets, and CPK biomarkers for their survival during their follow-up months.

## 12. Limitation

The experiments had been conducted on small dataset having confined features and instances. The patients under observations were above age of 40 years. Lack of real-time Implementation of the presented models must be include more samples and features to gain more significant accuracies of predictive models to make generalization for the whole population. The applied statistical and machine learning techniques were also confined. More feature selection algorithms approaches: Info gain, gain ratio, relief, and $\chi^{2}$ might be useful to improve the performance of the predictive models.

## 13. Conclusions

Wearable sensor technologies, particularly for chronic heart disease, can be used efficiently in the healthcare industry. Many lives can be saved by using monitoring and prediction systems, especially when the patient is in a remote location without access to medical care. This paper proposed a wearable PKI secured IoT enabled Smart Healthcare using a machine learning system based on the CDF-DI monitoring system. The present research confirmed the importance of Sodium and creatinine levels in the human body. It was revealed the significance of SC and SS towards the Survival-Status (Alive/Dead) of CVD patients (*p* < 0.05). We also found that the anemia level, High BP level, and diabetic level have no significant effect on the SC and SS biomarker levels (*p* > 0.05). Further, a significant association of smoking habits with specific patients towards gender was observed (*p* < 0.05). We conclude that the patients belonging to the very old age group were more mortality prone than adult patients. The platelets, CPK, SC, and EF were the four most prominent cardiac features that played their role well in the patient’s demography identification. In addition to the earlier study’s features EF, and SC [32,42], the authors recommended three more features: Platelets, CPK, and gender to identify the Survival-Status of the patient. This research achieved the highest prediction accuracy of 96% to predict the survival status of HF patients using the winner RF algorithm. The CDF-DI monitoring system would be a significant socio-economic health care system for cardiac patients after its implementation and integration. With early prediction using IoT enabled CDF-DI, patients can escape from the costlier health care tests or checkups. Positively, the present research work might be a milestone in the IoT enabled health care industry and help to save human lives from this severe cardiac disease.

## Figures and Tables

**Table 1:** Previous research versus Extant research.

Ref.	Tech.	DV	FS Algo.	CV-HP	Multcol.	IoT
[4]	IoT based 3-Tier Architecture, Wearable Sensors, Apache Hbase, Apache Mahout, LR	Heart Disease	×	×	×	*√*
[5]	Fuzzy Rule based Neural Network, Cloud and IoT based	Diabetes disease	×	×	×	*√*
[14]	T-Test, Fisher Exact Test	SC, Renal Insufficiency	×	×	×	×
[30]	Code free deep learning model	Gender	×	×	×	×
[31]	LR, K-NN, ANN, SVM (RBF), SVM (Linear), NB, DT, LOSOCV, Feature Selection	HD (Present/Absent)	Relief, MRMR, LASSO, LLBFS, FCMIM	LOSO	X	X
[32]	RF, DT, GBM, LR, ANN, NB, SVM (RBF), SVM (Linear), KNN, MCC	Survival Check (Survived/Dead)	RF	Grid Search	×	×
[33]	Stratified cox proportional Hazard regression model	4 Age-Group examined with Survival Analysis (time-to-event)	Statistical Analysis	×	×	×
[42]	COX Regression Model	EF levels (EF 45) with Survival (Time to event)	Statistical Analysis	X	X	X
[43]	SMOTE, DT, Ada-boost, LR, SGD, RF, GBM, ETC, NB, SVM	Survival Check (Survived/Dead)	RF	×	×	×
Present	Mann–Whitney *U*-Test, $\chi^{2}$ test, MS-AZURE, Raspberry pi4, Wearable Sensor medical devices, Cox Regression, DT, LR, GBM, GNB, RF, SVM (RBF), KNN, XGB, VIF, MCC, PKI	Survival-Status (Alive/Dead), Age-Group, Gender, SC and SS	RF, XGB	Grid Search	*√*	*√*

Source: Own elaboration.

**Table 2:** Dataset Description.

Continuous Variables				Categorical Variables		
**Attribute Name**	**Description**	**Range**	**Measured in**	**Attribute Name**	**Description**	**Range**
Platelets	Platelets in blood	25,100–85,000	kiloplatelets/mL	Gender	Woman/man	0–1
Age	Age of Patient	40–95	Years	Smoking	Yes/No	0–1
SS	135.39	114–148	mEq/L	Diabetes	40 (42%)	0–1
SC	Level of creatinine in the blood	0.50–9.40	mg/dL	High BP	Yes/No	0–1
EF	Percentage of leaving the heart at each concentration	14–80	Percentage	Anaemia	Decrease in Red Blood Cell/Haemoglobin	0–1
CPK	Level of CPK enzymes in the blood	23–7861	Mcg/L	Survival-Status	Died/Alive	0–1
Time	Follow up Month	4–285	Days			

Source: Own elaboration.

**Table 3:** Feature’s VIF of Gender and Age.

	Towards Gender		Towards Age	
**Features**	**VIF Score**	**VIF Score (Backward Elimination)**	**VIF Score**	**VIF Score (Backward Elimination)**
Age	30.12	Removed	30.40	Removed
Anaemia	1.90	1.79	1.91	1.79
CPK	1.45	1.40	1.46	1.42
Diabetes	1.78	1.75	1.79	1.75
EF	13.09	7.78	13.35	7.91
High BP	1.63	1.57	1.65	1.57
Platelets	8.49	7.02	8.64	7.02
SC	3.13	3.03	3.13	3.05
SS	58.37	Removed	61.55	Removed
Smoking	1.55	1.47	3.81	3.29
Time	5.65	4.02	1.89	1.89
Survival-Status	2.46	1.94	5.66	4.16
			2.47	1.97

Source: Own elaboration.

**Table 4:** Model Hyperparameter Tuning.

Classifier	Model Tuning Parameters
RF	criterion = ’gini’, max_features = 7, min_samples_leaf = 2, min_samples_split= 2, n_estimators=50
DT	criterion = ’gini’, max_depth = 50, max_features = ’log2’, min_samples_leaf = 1, min_samples_split = 50
SVM	C = 10, gamma = 0.001, kernel = ’rbf’
GBM	learning_rate = 0.001, max_depth = 3, n_estimators = 1000, subsample = 0.5
XGB	Gamma = 0, learning_rate = 0.1, max_delta_step = 2, max_depth = 6, min_child_weight = 4, n_estimators = 200, reg_alpha = 0, reg_lambda = 8
k-NN	metric = ’manhattan’, n_neighbors = 3, weights = ’uniform’
LR	C = 1.0, penalty = ’l2’, solver = ’newton–cg’

Source: Own elaboration.

**Table 5:** Proposed Hardware for CDF-DI System.

Hardware	Description
HeartGuideBP8000m	Omron Wearable smartwatch for BP monitoring
PC100-Platelet Counter	Point of Care Platelet/Thrombocyte Counting
Freestyle Libre Flash Glucose Monitoring System	Wearable Sensor, ASIN: B08M1CMWZW
Raspberry Pi4	1.5 Ghz quad core 64 bit ARM cortex A-72CPU
Personal Computer/Laptop	Intel® coreTM i3 processor
Nova StatSensor Creatinine	Portable, Biosensor Blood Creatinine Analyzer/Miniaturized

**Table 6:** Machine Learning Models.

Model	Description	Reference
DT	DT is an algorithm of classification which works well on categorical and numerical forms of data. It is generally used to build tree-like structures. Medical data can be analysed easily with good accuracy.	[54]
RF	RF is a model of tree-based ensemble learning that produces exact prediction by combining several weak learners. This model uses the bagging technique for training a range of decision tree with different bootstrap samples	[43]
k-NN	When compared to a collection of known data, the k-NN method allows us to identify unknown data by calculating the distance or similarity of an unknown datum. It assigns a class to the datum based on the number of neighbors with the same class who are the nearest to it. k controls or indicates the number of neighbors used in the decision.	[46]
LR	LR typically predictive analysis based on the concept of probability. Binary categorical variable is predicted by one or more independent variable using sigmoid function	[55]
XGB	The XGB is a popular ensemble learning algorithm that uses DT models in the background for computation. It is a highly effective scalable machine learning algorithm. It combines multiple weak-learner to build a strong classifier proved a better classifier.	[56]
GBM	Many weak classifiers work together to build a powerful model for learning on the GBM. It usually time taking process due to creation of many independent tree. It has ability to deal with missing values	[57]
GNB	GNB is a naive bayes variant that works with gaussian distributions and is used for continuous data. The prior and posterior likelihood of the class in the data are involved in conjunction with a function that has constant values. All of the features are often assumed to obey a gaussian or regular distribution	[58]
SVM	SVM is a mathematical model-based supervised learning technique. It is used to solve problems including regression and classification problems. It classifies data by creating high-dimensional hyperplanes, also known as decision planes. Hyper planes are used to separate one form of data from another	[59]

Source: Own elaboration.

**Table 7:** Impact of SC and SS on Survival-Status of the Patient.

Parameter	Distribution Normal	Homogeneity in Variance	μ Rank	*U*	Sig. 2-Tailed (*p*)
SC	×	×	0:128.10	5298.00	0.00 *
			1:196.31		
SS	×	*√*	0:162.40	7226.50	0.00 *
			1:123.78		

Source: Own elaboration. * is 0.05 significance level.

**Table 8:** Impact of SC and SS w.r.t Anemic, Diabetic and BP levels.

Variable & Assumptions			Anemic Levels			Diabetic Levels			BP Levels		
**Parameter**	**Distribution** **Normal**	**Homogeneity** **in Variance**	μ **Rank**	*U*	***p***	μ **Rank**	***U***	***p***	μ Rank	***U***	***p***
SC	×	*√*	0:151.22	10,758	0.779	0:149.86	10,850.0	0.973	0:155.67	9085	0.122
			1:148.40			1:150.20			1:139.52		
SS	×	*√*	0:145.40	10183.5	0.289	0:154.03	10,173.5	0.339	0:148.78	9948.50	0.739
			1:156.06			1:144.38			1:152.25		

Source: Own elaboration.

**Table 9:** Observed value of Gender vs. Smoking Status.

Gender	Non-Smoker	Smoker	Row Marginals (Row Sum)
Female	101	4	**105**
Male	102	92	**194**
Column Marginals (Columns Sum)	**203**	**96**	**299**

Source: Own elaboration.

**Table 10:** Expected value of Gender vs. Smoking Status ($\chi^{2}$ Values).

Gender	Non-Smoker	Smoker	*df*	$\chi^{2}$	*p*
Female	71.30 (12.37)	33.7 (26.17)	1	59.45	0.00 *
Residual	29.7	−29.7			
Male	131.70 (6.70)	62.3 (14.16)			
Residual	−29.7	29.7			

Source: Own elaboration. * is 0.05 significance level.

**Table 11:** Age group vs. Survival-Status Descriptive Summary.

Case Processing Summary				
**Age_Group**	**Total Patients**	**No. of Deaths**	**Censored**	
			***N***	**Percent**
Adult	129	31	98	76.0%
Very Old	170	65	105	61.8%
Overall	299	96	203	67.9%

Source: Own elaboration.

**Table 12:** Significance Check towards Survival-Status.

Variable	β Coefficient	HR	*p*
Anemia	0.497	1.644	0.022 *
CPK	0.000	1.0	0.020 *
Diabetes	−0.055	0.946	0.800
EF	−0.046	0.955	0.000 *
high_BP	0.490	1.632	0.023 *
Platelets	0.000	1.0	0.972
Gender	−0.134	0.875	0.590
smoking	0.058	1.059	0.818
Age-Group	−0.596	0.551	0.008 *

Source: Own elaboration. * is 0.05 significance level.

**Table 13:** Gender Classification Performance Metrics.

Classifier	MCC	F1-Score	Accuracy	Recall (TPR)	Precision	TNR
MCC Ranking:						
RF	**+0.87 ± 0.25**	0.95 ± 0.09	0.94 ± 0.11	0.95 ± 0.11	0.95 ± 0.08	0.91 ± 0.14
GBM	**+0.31 ± 0.19**	0.71 ± 0.06	0.65 ± 0.08	0.64 ± 0.08	0.79 ± 0.09	0.68 ± 0.17
LR	**+0.25 ± 0.14**	0.75 ± 0.04	0.67 ± 0.05	0.78 ± 0.08	0.73 ± 0.05	0.45 ± 0.14
Linear SVM	**+0.24 ± 0.13**	0.75 ± 0.04	0.66 ± 0.05	0.78 ± 0.07	0.72 ± 0.05	0.45 ± 0.13
DT	**+0.22 ± 0.20**	0.72 ± 0.06	0.64 ± 0.07	0.72 ± 0.13	0.73 ± 0.08	0.50 ± 0.24
XGB	**+0.21 ± 0.18**	0.74 ± 0.06	0.65 ± 0.08	0.76 ± 0.09	0.72 ± 0.04	0.45 ± 0.10
K-NN	**+0.12 ± 0.11**	0.73 ± 0.04	0.62 ± 0.05	0.80 ± 0.08	0.68 ± 0.04	0.30 ± 0.11
GNB	**+0.06 ± 0.16**	0.71 ± 0.05	0.59 ± 0.06	0.76 ± 0.09	0.66 ± 0.04	0.29 ± 0.12
F1-Score Ranking:						
RF	+0.87 ± 0.25	**0.95 ± 0.09**	0.94 ± 0.11	0.95 ± 0.11	0.95 ± 0.08	0.91 ± 0.14
LR	+0.25 ± 0.14	**0.75 ± 0.04**	0.67 ± 0.05	0.78 ± 0.08	0.73 ± 0.05	0.45 ± 0.14
SVM	+0.24 ± 0.13	**0.75 ± 0.04**	0.66 ± 0.05	0.78 ± 0.07	0.72 ± 0.05	0.45 ± 0.13
XGB	+0.21 ± 0.18	**0.74 ± 0.06**	0.65 ± 0.08	0.76 ± 0.09	0.72 ± 0.04	0.45 ± 0.10
k-NN	+0.12 ± 0.11	**0.73 ± 0.04**	0.62 ± 0.05	0.80 ± 0.08	0.68 ± 0.04	0.30 ± 0.11
DT	+0.22 ± 0.20	**0.72 ± 0.06**	0.64 ± 0.07	0.72 ± 0.13	0.73 ± 0.08	0.50 ± 0.24
GBM	+0.31 ± 0.19	**0.71 ± 0.06**	0.65 ± 0.08	0.64 ± 0.08	0.79 ± 0.09	0.68 ± 0.17
GNB	+0.06 ± 0.16	**0.71 ± 0.05**	0.59 ± 0.06	0.76 ± 0.09	0.66 ± 0.04	0.29 ± 0.12
Accuracy Ranking:						
RF	+0.87 ± 0.25	0.95 ± 0.09	**0.94 ± 0.11**	0.95 ± 0.11	0.95 ± 0.08	0.91 ± 0.14
LR	+0.25 ± 0.14	0.75 ± 0.04	**0.67 ± 0.05**	0.78 ± 0.08	0.73 ± 0.05	0.45 ± 0.14
SVM	+0.24 ± 0.13	0.75 ± 0.04	**0.66 ± 0.05**	0.78 ± 0.07	0.72 ± 0.05	0.45 ± 0.13
XGB	+0.21 ± 0.18	0.74 ± 0.06	**0.65 ± 0.08**	0.76 ± 0.09	0.72 ± 0.04	0.45 ± 0.10
GBM	+0.31 ± 0.19	0.71 ± 0.06	**0.65 ± 0.08**	0.64 ± 0.08	0.79 ± 0.09	0.68 ± 0.17
DT	+0.22 ± 0.20	0.72 ± 0.06	**0.64 ± 0.07**	0.72 ± 0.13	0.73 ± 0.08	0.50 ± 0.24
k-NN	+0.12 ± 0.11	0.73 ± 0.04	**0.62 ± 0.05**	0.80 ± 0.08	0.68 ± 0.04	0.30 ± 0.11
GNB	+0.06 ± 0.16	0.71 ± 0.05	**0.59 ± 0.06**	0.76 ± 0.09	0.66 ± 0.04	0.29 ± 0.12

Source: Own elaboration.

**Table 14:** Gender classifier’s CMs.

Model		GBM		k-NN		RF		DT		LR		GNB		XGB		SVM	
Predicted																	
Actual	Gender	M	W	M	W	M	W	M	W	M	W	M	W	M	W	M	W
	M	125	69	155	39	185	9	139	55	152	42	147	47	147	47	152	42
	W	34	71	32	73	9	96	52	53	57	48	74	31	58	47	58	47

Source: Own elaboration.

**Table 15:** Age Group Classification Performance Metrics.

Classifier	MCC	F1-Score	Accuracy	Recall		
(TPR)	Precision	TNR				
MCC Ranking:						
RF	**+0.92 ± 0.23**	0.96 ± 0.09	0.96 ± 0.11	0.97 ± 0.07	0.95 ± 0.10	0.95 ± 0.16
GBM	**+0.25 ± 0.14**	0.73 ± 0.05	0.64 ± 0.05	0.86 ± 0.10	0.64 ± 0.04	0.35 ± 0.13
DT	**+0.23 ± 0.11**	0.68 ± 0.07	0.62 ± 0.05	0.69 ± 0.14	0.66 ± 0.06	0.53 ± 0.17
XGB	**+0.23 ± 0.15**	0.67 ± 0.07	0.59 ± 0.07	0.64 ± 0.10	0.68 ± 0.06	0.56 ± 0.11
LR	**+0.16 ± 0.13**	0.69 ± 0.03	0.58 ± 0.05	0.77 ± 0.05	0.62 ± 0.06	0.37 ± 0.15
SVM	**0.12 ± 0.14**	0.67 ± 0.05	0.58 ± 0.06	0.73 ± 0.09	0.61 ± 0.06	0.38 ± 0.14
k-NN	**+0.09 ± 0.11**	0.62 ± 0.08	0.56 ± 0.05	0.63 ± 0.14	0.61 ± 0.05	0.46 ± 0.14
GNB	**0.02 ± 0.17**	0.68 ± 0.03	0.55 ± 0.05	0.85 ± 0.07	0.57 ± 0.05	0.17 ± 0.15
F1-Score Ranking:						
RF	+0.92 ± 0.23	**0.96 ± 0.09**	0.96 ± 0.11	0.97 ± 0.07	0.95 ± 0.10	0.95 ± 0.16
GBM	+0.25 ± 0.14	**0.73 ± 0.05**	0.64 ± 0.05	0.86 ± 0.10	0.64 ± 0.04	0.35 ± 0.13
LR	+0.16 ± 0.13	**0.69 ± 0.03**	0.58 ± 0.05	0.77 ± 0.05	0.62 ± 0.06	0.37 ± 0.15
GNB	0.02 ± 0.17	**0.68 ± 0.03**	0.55 ± 0.05	0.85 ± 0.07	0.57 ± 0.05	0.17 ± 0.15
DT	+0.23 ± 0.11	**0.68 ± 0.07**	0.62 ± 0.05	0.69 ± 0.14	0.66 ± 0.06	0.53 ± 0.17
SVM	0.12 ± 0.14	**0.67 ± 0.05**	0.58 ± 0.06	0.73 ± 0.09	0.61 ± 0.06	0.38 ± 0.14
XGB	+0.23 ± 0.15	**0.67 ± 0.07**	0.59 ± 0.07	0.64 ± 0.10	0.68 ± 0.06	0.56 ± 0.11
K-NN	+0.09 ± 0.11	**0.62 ± 0.08**	0.56 ± 0.05	0.63 ± 0.14	0.61 ± 0.05	0.46 ± 0.14
Accuracy Ranking:						
RF	+0.92 ± 0.23	0.96 ± 0.09	**0.96 ± 0.11**	0.97 ± 0.07	0.95 ± 0.10	0.95 ± 0.16
GBM	+0.25 ± 0.14	0.73 ± 0.05	**0.64 ± 0.05**	0.86 ± 0.10	0.64 ± 0.04	0.35 ± 0.13
DT	+0.23 ± 0.11	0.68 ± 0.07	**0.62 ± 0.05**	0.69 ± 0.14	0.66 ± 0.06	0.53 ± 0.17
XGB	+0.23 ± 0.15	0.67 ± 0.07	**0.59 ± 0.07**	0.64 ± 0.10	0.68 ± 0.06	0.56 ± 0.11
LR	+0.16 ± 0.13	0.69 ± 0.03	**0.58 ± 0.05**	0.77 ± 0.05	0.62 ± 0.06	0.37 ± 0.15
SVM	0.12 ± 0.14	0.67 ± 0.05	**0.58 ± 0.06**	0.73 ± 0.09	0.61 ± 0.06	0.38 ± 0.14
k-NN	+0.09 ± 0.11	0.62 ± 0.08	**0.56 ± 0.05**	0.63 ± 0.14	0.61 ± 0.05	0.46 ± 0.14
GNB	0.02 ± 0.17	0.68 ± 0.03	**0.55 ± 0.05**	0.85 ± 0.07	0.57 ± 0.05	0.17 ± 0.15

Source: Own elaboration.

**Table 16:** Age group classifier’s CMs.

Model		GBM		k-NN		RF		DT		LR		GNB		XGB		SVM	
Predicted																	
Actual	Age Group	A	VO	A	VO	A	VO	A	VO	A	VO	A	VO	A	VO	A	VO
	A	45	84	59	70	122	7	69	60	46	83	22	107	72	57	49	80
	VO	23	147	62	108	5	165	52	118	39	131	26	144	55	115	45	125

Source: Own elaboration.

**Table 17:** Survival-Status Classification Performance Metrics.

Classifier	MCC	F1-Score	Accuracy	Recall (TPR)	Precision	TNR
MCC Ranking:						
RF	**+0.91 ± 0.11**	0.94 ± 0.07	0.96 ± 0.05	0.93 ± 0.08	0.95 ± 0.08	0.97 ± 0.05
DT	**+0.63 ± 0.11**	0.75 ± 0.07	0.83 ± 0.05	0.80 ± 0.10	0.71 ± 0.09	0.85 ± 0.06
XGB	**+0.63 ± 0.12**	0.74 ± 0.10	0.84 ± 0.05	0.72 ± 0.17	0.77 ± 0.10	0.90 ± 0.05
LR	**+0.59 ± 0.10**	0.71 ± 0.08	0.82 ± 0.04	0.66 ± 0.11	0.77 ± 0.09	0.91 ± 0.04
GBM	**+0.59 ± 0.14**	0.72 ± 0.10	0.83 ± 0.05	0.69 ± 0.15	0.75 ± 0.12	0.89 ± 0.06
GNB	**+0.53 ± 0.17**	0.64 ± 0.15	0.81 ± 0.06	0.54 ± 0.17	0.79 ± 0.14	0.93 ± 0.05
SVM	**+0.13 ± 0.14**	0.21 ± 0.11	0.68 ± 0.03	0.13 ± 0.08	0.52 ± 0.24	0.94 ± 0.03
k-NN	**+0.06 ± 0.16**	0.33 ± 0.12	0.61 ± 0.06	0.30 ± 0.12	0.37 ± 0.14	0.77 ± 0.07
F1-Score Ranking:						
RF	+0.91 ± 0.11	**0.94 ± 0.07**	0.96 ± 0.05	0.93 ± 0.08	0.95 ± 0.08	0.97 ± 0.05
DT	+0.63 ± 0.11	**0.75 ± 0.07**	0.83 ± 0.05	0.80 ± 0.10	0.71 ± 0.09	0.85 ± 0.06
XGB	+0.63 ± 0.12	**0.74 ± 0.10**	0.84 ± 0.05	0.72 ± 0.17	0.77 ± 0.10	0.90 ± 0.05
GBM	+0.59 ± 0.14	**0.72 ± 0.10**	0.83 ± 0.05	0.69 ± 0.15	0.75 ± 0.12	0.89 ± 0.06
LR	+0.59 ± 0.10	**0.71 ± 0.08**	0.82 ± 0.04	0.66 ± 0.11	0.77 ± 0.09	0.91 ± 0.04
GNB	+0.53 ± 0.17	**0.64 ± 0.15**	0.81 ± 0.06	0.54 ± 0.17	0.79 ± 0.14	0.93 ± 0.05
k-NN	+0.06 ± 0.16	**0.33 ± 0.12**	0.61 ± 0.06	0.30 ± 0.12	0.37 ± 0.14	0.77 ± 0.07
SVM	+0.13 ± 0.14	**0.21 ± 0.11**	0.68 ± 0.03	0.13 ± 0.08	0.52 ± 0.24	0.94 ± 0.03
Accuracy Ranking:						
RF	+0.91 ± 0.11	0.94 ± 0.07	**0.96 ± 0.05**	0.93 ± 0.08	0.95 ± 0.08	0.97 ± 0.05
XGB	+0.63 ± 0.12	0.74 ± 0.10	**0.84 ± 0.05**	0.72 ± 0.17	0.77 ± 0.10	0.90 ± 0.05
DT	+0.63 ± 0.11	0.75 ± 0.07	**0.83 ± 0.05**	0.80 ± 0.10	0.71 ± 0.09	0.85 ± 0.06
GBM	+0.59 ± 0.14	0.72 ± 0.10	**0.83 ± 0.05**	0.69 ± 0.15	0.75 ± 0.12	0.89 ± 0.06
LR	+0.59 ± 0.10	0.71 ± 0.08	**0.82 ± 0.04**	0.66 ± 0.11	0.77 ± 0.09	0.91 ± 0.04
GNB	+0.53 ± 0.17	0.64 ± 0.15	**0.81 ± 0.06**	0.54 ± 0.17	0.79 ± 0.14	0.93 ± 0.05
SVM	+0.13 ± 0.14	0.21 ± 0.11	**0.68 ± 0.03**	0.13 ± 0.08	0.52 ± 0.24	0.94 ± 0.03
k-NN	+0.06 ± 0.16	0.33 ± 0.12	**0.61 ± 0.06**	0.30 ± 0.12	0.37 ± 0.14	0.77 ± 0.07

Source: Own elaboration.

**Table 18:** Survival Status classifier’s CMs.

Model		GBM		k-NN		RF		DT		LR		GNB		XGB		SVM	
Predicted																	
Actual	Survival-Status	D	AL	D	AL	D	AL	D	AL	D	AL	D	AL	D	AL	D	AL
	D	66	30	29	67	89	7	77	19	63	33	52	44	69	27	54	42
	AL	22	181	49	154	5	198	31	172	20	183	14	189	21	182	13	190

Source: Own elaboration.

**Table 19:** Benchmark with previous study results.

CDF-DI (Extant Research)						Davide Chicco et al. [32]					
**Model**	**Accuracy**	**F1-Score**	**MCC**	**Sen.**	**Spec.**	**Existing** **Accuracy**	**Existing** **F1-Score**	**Existing** **MCC**	**Sen.**	**Spec.**	**Extant** **Accuracy**
RF	**0.96**	0.94	0.91	0.93	0.97	0.74	**0.55**	0.38	0.49	0.86	0.22 ↑
DT	**0.83**	0.75	0.63	0.80	0.97	0.74	**0.55**	0.38	0.53	0.83	0.09 ↑
GBM	**0.83**	0.72	0.59	0.69	0.89	0.74	**0.53**	0.37	0.48	0.86	0.09 ↑
LR	**0.82**	0.71	0.59	0.66	0.91	0.73	**0.47**	0.33	0.39	0.89	0.09 ↑
GNB	**0.81**	0.64	0.53	0.54	0.93	0.70	**0.36**	0.22	0.28	0.90	0.11 ↑
SVM	**0.68**	0.21	0.13	0.13	0.94	0.69	**0.18**	0.16	0.12	0.97	−0.01↓
k-NN	**0.61**	0.33	0.06	0.30	0.77	0.62	**0.15**	−0.02	0.12	0.87	−0.01↓

Source: Own elaboration.

## Data Availability

Data is available on UCI Machine Learning repository.

## Abbreviations

The following abbreviations are used in this manuscript:

IoT	Internet of things
CVD	CardioVascular Diseases
HF	Heart Failures
HD	Heart Disease
SC	Serum Creatinine
SS	Serum Sodium
EF	Ejection Fraction
CPK	Creatinine Phosphokinase
BP	Blood-Pressure
DT	Decision Tree
RF	Random Forest
SD	Standard Deviation
SVM	Support Vector Machine
KNN	k-Nearest Neighbors
XGB	eXtreme Gradient Boosting
GBM	Gradient Boosting Machines
LR	Logistic Regression
LNR	Linear Regression
AUC	Area Under Curve
VIF	Variation Inflation Factor
GNB	Gaussian Naive Bayes
Mdn	Median
TPR	True Positive Rate
TNR	True Negative Rate
CMs	Confusion matrics
PKI	Public Key Infrastructure
